# Can Reactive School Closures help critical care provision during the current influenza pandemic?

**DOI:** 10.1371/currents.RRN1119

**Published:** 2009-10-23

**Authors:** Thomas House, Marc Baguelin, Albert Jan van Hoek, Stefan Flasche, P White, Md Zia Sadique, Ken Eames, Jonathan Read, Niel Hens, Alessia Melegaro, Matt Keeling

**Affiliations:** ^*^Department of Biological Sciences and Mathematics Institute, University of Warwick, Coventry, CV4 7AL, UK.; ^†^Health Protection Agency, London, UK; ^‡^Health Protection Agency; ^§^Health Protection Agency, London, UK / University of Strathclyde, Glasgow, UK; ^¶^HPA; ^#^London School of Hygiene and Tropical Medicine, Keppel Street, London, WC1E 7HT; ^**^Epidemiology group & National Centre for Zoonosis Research, Faculty of Veterinary Science, University of Liverpool, Leahurst Campus, The Wirral, CH64 7TE, UK; ^††^I-Biostat, Hasselt University, Belgium & CHERMID, CEV, Vaxinfectio, University of Antwerp, Belgium; ^‡‡^Dondena Centre for Research on Social Dynamics Bocconi University and ^§§^Biological Sciences & Mathematics Institute, University of Warwick, Coventry, CV4 7AL, UK

## Abstract

Although the current H1N1 influenza strain is now considered to be relatively mild, it still has the potential to place a serious burden on health services. The closure of schools has been postulated as a means of reducing transmission between children and hence reducing the number of cases at the peak of an epidemic; however if instigated nationally such a policy has severe economic costs. Reactive short-duration closure of schools in regions where health services are close to capacity offers a potential compromise, but it is unclear over what spatial scale and timeframe closures would need to be made to have a substantial impact. Here, using detailed geographic information for England, we assess how localized school closures could alleviate the burden on hospital intensive care units (ICUs) that are reaching capacity. We show that, for a range of epidemiologically plausible assumptions, considerable local coordination of school closures is needed to achieve a substantial reduction in the number of hospitals that are over capacity at the epidemic peak. The heterogeneity in demand per hospital means that even widespread school closures are unlikely to impact on whether demand will exceed capacity for many hospital ICUs. These results re-enforce the UK policy of not utilising school closures as a control mechanism, but have far wider international public-health implications. The spatial heterogeneities in both population density and hospital capacity that give rise to our results are present in many Northern Hemisphere countries where a second wave of influenza is predicted this autumn and winter. This leads us to believe that even widespread reactive school closures are unlikely to eliminate problems of demand exceeding local capacity in many regions.

## Introduction

The current outbreak of H1N1 influenza was first reported in Mexico in April 2009, and initial reports suggested that it was associated with a large proportion of case fatalities [1]. On 28 April the first cases were confirmed in the UK, and on 11 June the WHO officially declared a pandemic. Data from a variety of sources, in particular the Southern Hemisphere where the winter 'flu season is at an end, has shown that this strain is associated with far lower mortality than first anticipated and is most prevalent in children - the elderly, who usually suffer the most adverse reactions to influenza, appear to have a much lower infection risk [2]. However, despite the fact that this infection appears relatively mild in comparison to previous influenza pandemics (particularly the 1918 outbreak) it is still likely to place a considerable burden upon healthcare services. Intensive care units are likely to come under particular pressure near the peak of the epidemic that is expected later this year. Two options therefore can be considered to alleviate this burden: either increasing ICU capacity or decreasing the epidemic peak. Recent English Department of Health plans aim to double the number of adult intensive care beds available in an effort to mitigate the worst effects [3], although given uncertainties in epidemic predictions and heterogeneity in hospital capacity there are still likely to be some regions where peak demand exceeds capacity. 

 School closures have been proposed as a viable method of reducing both the size and peak of an epidemic [4]. Data from a variety of previous epidemics suggests that a significant proportion of seasonal influenza transmission occurs within schools, such that closing schools may reduce the peak by as much as 45% [5]. Given that the current H1N1 strain is more prevalent in children than is usually the case for seasonal influenza, there is the potential that an even greater reduction could be achieved. This potential impact is supported by data from England: during June and early July cases were doubling approximately every week, however during the school holidays estimated cases dropped by around 40% each week [2].  Although these data are influenced by reporting biases and the impact of family vacations, they suggest the potential power of school closures. However, such school closures have severe implications both economically and in terms of public-health services due to the number of working parents (especially health care workers) that would be forced to take time off. For these reasons national school closures are an unlikely strategy in the current outbreak, but small-scale reactive closures could be considered as a means of reducing the peak incidence in regions where hospitals are reaching capacity.  It is this reactive use of school closures that we investigate here. When considering hospital capacity, we focus on adult ICU facilities for two reasons: firstly, ICU beds are likely to be closer to capacity than standard hospital beds; and secondly, paediatric ICUs are sparser geographically than adult ICUs, meaning that long-distance transfers and use of adult ICUs as appropriate are already integral to NHS strategy for children [3].

## Methods

 To gauge the approximate reduction in the epidemic peak that can be achieved through school closures we utilize a standard age-structured epidemic model, with between age-group mixing derived from the pan-European POLYMOD survey [6] [7] and age-dependent susceptibility parameterized to match age-stratified prevalence and early epidemic growth rates (see Supplementary Material). This model provides a good description of both the early dynamics of infection in England and also the decline in cases during the school holidays.

 Given the range of uncertainty in many of the fundamental parameters, when considering the impact of school closures on capacity we adopt a parsimonious approach in which we assume that the peak national demand on adult ICU facilities and reduction from school closures are static model parameters chosen within suitable ranges.  For each hospital where the peak demand on adult ICU beds is above capacity we consider two strategies: either individual local schools are shut sequentially in the order that maximizes the impact on the associated hospital; or all the schools located in nearby LAs are shut, with the sequence of LAs affected determined by the order that maximizes the impact on the associated hospital. In practice this means first implementing closures of schools, or in LAs, that have the greatest population overlap between school and hospital catchment areas. To determine the total distances moved to critical care beds, we use a Monte Carlo algorithm in which patients are randomly permuted and assigned to the available ICU bed nearest their local hospital.

## Results

 We simulate the impact of a variety of school closures from 1 to 4 weeks in total at a variety of points during the predicted Autumn/Winter epidemic; the effect of school closures on both the peak daily incidence and the total number of cases is shown in Figure 1.  Longer closures, multi-interval closures and closures starting before the peak of the epidemic have the greatest impact on the predicted peak incidence, while later closures have a greater effect on the total number of cases. Although this figure illustrates that a substantial reduction in the peak is possible, the magnitude of this reduction is highly sensitive to the timing of closures.  This means that significant coordination of closures would be required for them to have the maximal desired effect.

 Figure 2 focuses on the overlapping geography associated with local administration of schools, primary care, and hospitals in England. Hospitals are shown as points, colour-coded by their capacity (total beds in Pane 2(a) and adult ICU beds in Pane 2(b)); as expected, hospitals are aggregated in regions of high population density. In Pane 2(a), delineated regions correspond to local authorities (LAs) who have responsibility for local schools, while coloured regions correspond to Primary Care Trusts (PCT) who are responsible for local primary care - in the majority of cases each PCT contains multiple LAs. Catchment areas for each hospital, based on shortest distance (Pane 2(b)) cannot be simply related to either of the two administrative units shown in Pane 2(a). The spatial arrangement of hospitals, together with a policy of assignment to the closest hospital, leads to considerable heterogeneity in the number of individuals within the catchment area relative to the number of general or ICU beds, with no clear spatial pattern to this variability - furthermore, alternative localised methods of assigning patients to hospitals do not substantially diminish this heterogeneity. These results suggest that even if there is sufficient national capacity to deal with the current influenza outbreak, many hospitals and their ICUs could be overwhelmed - an effect which could potentially be exacerbated if some regions experience substantially higher epidemic peaks than the average.

 Faced with this lack of capacity in some hospitals, we consider how closing schools and thereby reducing the local epidemic peak could be used to reduce the number of ICUs with excess demand, and we assess the scale at which such closures would need to be enacted.  Three measures are used to capture the impact of these closures (Figure 3): the percentage of hospitals where adult ICU demand from the local catchment area still exceeds capacity (Panes 3(a) and 3(b)); the maximum number of adults requiring ICU above local capacity (Panes 3(b) and 3(c)); and the total additional distance travelled if adults are moved from their primary hospital to the nearest secondary hospital with spare ICU capacity (Panes 3(d) and 3(e)). 

 Considering the scenario where, in the absence of school closures, peak national demand equals national capacity (solid lines) we observe that 60% of hospitals have a local demand that exceeds their capacity. Even with optimistic assumptions about school closures (reducing the peak demand by 60%, blue line), this cannot be brought to zero and only achieves its lowest value of 12% above capacity when there is a school-level coordinated closure of at least 30% of all English schools or 50% of LAs. While this coordinated closure still leaves 12% of hospitals above capacity it does substantially reduce the amount by which capacity is exceeded in these regions and also reduces the distance patients needing ICU facilities have to be moved. Alternative, less optimistic assumptions concerning school closures (green and red lines) correspondingly have a more limited impact.

## Discussion

 The decision to close schools is clearly a trade-off between a variety of conflicting factors. Epidemiologically, social distancing measures such as school closures should reduce the scale of an epidemic and therefore provide a benefit to hospitals; however they also have substantial economic and social impacts [4] [8], and place additional pressures on business and health-care services that may already be struggling with absenteeism due to illness. Due to the large number of healthcare workers with responsibilities for children of school age, the rate of absenteeism due to school closure could exceed 30% [8]. In a situation where school closures are implemented near the epidemic peak for optimal effect, and where the potential capacity benefits of staff redeployment are already fully realised, this absenteeism rate would effectively discount the first 30\% of benefits in peak reduction due to school closure.

 Our results, derived using consistently optimistic assumptions about the impact of school closures, demonstrate that even locally reactive school closures would require a vast degree of coordination to mitigate the spatial heterogeneity in hospital capacity. It is likely that individual schools may need to close due to the impact of the epidemic on staffing levels, however these closures are unlikely to be sufficiently coordinated to offer protection to the surrounding population. While these results are based on the English spatial pattern of hospitals, schools and population density, we expect these results to be widely applicable due to the general difference in scales between hospital and school catchment areas and the ubiquitous heterogeneity in population density and hospital capacity.  Precise predictions of hospital demand and the impact of school closures for other countries will be critically dependent on public-health measures and a number of social factors; however our conclusions hold for a wide range of parameter assumptions and conclusions are therefore generally robust.  The only scenario under which school closures could be seen as a viable policy is when peak national demand (or peak demand in large-scale regions) exceeds total capacity - a reasonable worse-case prediction shown as the top, dashed, lines in Figure 3 - in which case a synchronized and sustained closure of schools could become necessary.

## Figures

**Figure:**
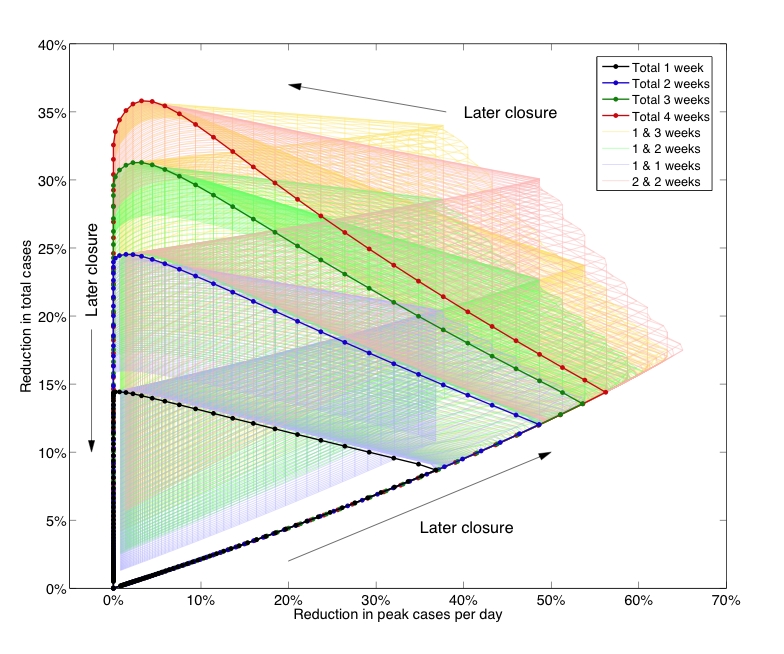
Figure 1: Impact of duration and timing on efficacy of school closure.  Policies of one continuous period of closure between one and four weeks are shown as thick lines. Closures are assumed to occur for whole days, and this daily interval is shown using dots that represent different timings of initial closure. All policies involving two periods of closure of one, two or three weeks summing to four weeks or less are shown as meshes. The strategies with first closure happening after the national peak all exhibit 0% reduction in the peak.

**Figure:**
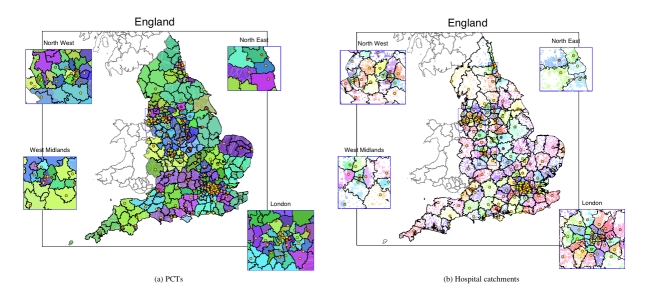
Figure 2: Relationship between different administrative units and hospital capacity. Local Authorities (LAs) are shown using grey lines for Scotland and Wales in both panes.  In Pane (a), LAs are shown using black lines for England, Primary Care Trusts (PCTs) are shown by coloured regions, and English NHS Trusts are shown by a circle at the location of the main hospital, with red indicating a larger number of total beds and yellow indicating a smaller number of total beds.  In Pane (b), English NHS Trusts with non-zero adult ICU capacity are shown by a circle at the location of the main hospital, with red indicating a larger number of adult ICU beds and yellow indicating a smaller number of adult ICU beds. The assumed catchment areas for these hospitals are shown using black lines containing coloured dots located at the population-weighted centroids of Lower Super Output Areas (LSOAs) to indicate population density.

**Figure:**
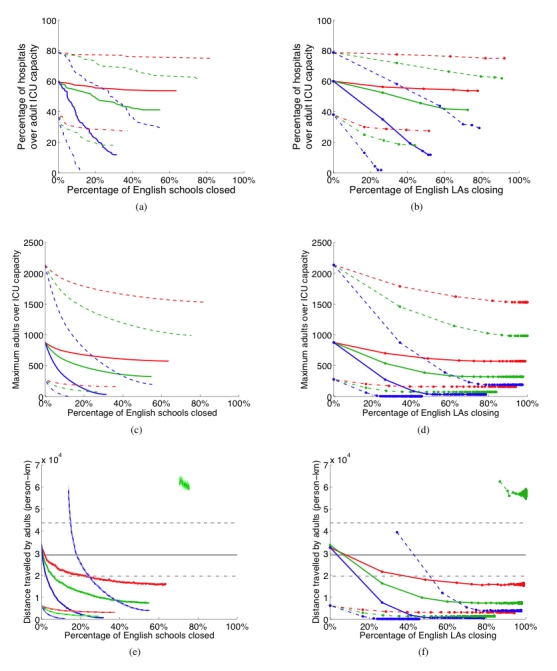
Figure 3: Effect of school closures on capacity. Red lines represent the scenario where school closures can reduce the local peak by 15%, green lines 30% and blue lines 60%. From top to bottom, in each graph, the series of lines represent different levels of national peak adult ICU demand as a percentage of adult ICU demand relative to national capacity: 150% (dashed); 100% (solid); and 67% (dash-dotted).  For Panes (e) and (f), grey lines represent distances travelled to the nearest hospital, with coloured lines the secondary distance moved to reach the nearest available ICU - an absence of a coloured line indicates national overcapacity. Figures in the left-hand column represent school-level targeting of closure, and in the right-hand column represent LA-level closures.

## Acknowledgements 

 We thank Martin Hensher, David Halsall and Ian Mills at the Department of Health for discussions during the preparation of this work. We thank Phil Sansom for help with latest PCT boundaries. We thank Sam Mason for help with data processing. 

## Funding Information

 This work was funded by: the Medical Research Council grant number G0701256 (http://www.mrc.ac.uk); the Economic and Social Research Council (http://www.esrc.ac.uk); the European Union FLUMODCONT project FP7-GA-201601 (http://www.flumodcont.eu); we thank the SIMID project number 060081 funded by the Institute for the Promotion of Innovation by Science and Technology in Flanders, IWT (http://www.iwt.be).  The funders had no role in study design, data collection and analysis, decision to publish, or preparation of the manuscript.

## Competing Interests

 The authors have declared that no competing interests exist.

## Supplementary Material

 We present here information that allows reproduction of our results, details the models used and assesses the sensitivity and validation of our assumptions.

### Dynamical model

The ODE model used to consider timing school closures in the main paper uses SEIR compartmental structure with gamma-distributed latent and recovery periods. In line with the observed serial interval of 3.7 days, we take transition rates as below.

\begin{equation*}E_1 \rightarrow E_2 \text{ at rate 1 days}^{-1}\end{equation*} \begin{equation*}E_2 \rightarrow I_1 \text{ at rate 1 days}^{-1}\end{equation*} \begin{equation*}I_1 \rightarrow I_2 \text{ at rate 1/(1.7) days}^{-1}\end{equation*} \begin{equation*}I_2 \rightarrow R \text{ at rate 1/(1.7) days}^{-1}\end{equation*}

The model is age-structured, with 7 age classes: 0; 1-4; 5-14; 15-24; 25-44; 45-64; 65+. Mixing matrices for the periods with open and closed schools are taken from the POLYMOD study, with transmission scaled to give reproduction numbers \begin{equation*}\mathcal{R}=1.44\end{equation*} prior to the summer holidays and \begin{equation*}\mathcal{R}=0.7\end{equation*} during. This represents an optimistic assumption about the impact of school closures, since the mixing matrices on their own do not predict such a significant reduction, meaning that family holidays and other factors peculiar to the summer are also likely to be reducing transmission. Age-stratified susceptibilities are determined from early age-specific attack rates. Figure S1 shows that model this agrees with the progress of the H1N1 pandemic in England to date. Detailed prediction of the timing and scale of the secondary peak is subject to many statistical uncertainties, and therefore the long-term dynamics of this model are not intended as definitive forecasts.

### Interaction between local areas

Our model for the impact of school closures assumes a linear relationship between the proportion of a hospital catchment population affected by school closure and the reduction in peak demand for adult ICU.  Model results relevant for this assumption are shown in Figure S2. Pane (a) shows the distances associated with work movement of adults (recorded at Output Area level in the 2001 UK census), together with age-stratified movements to school recorded at LSOA level for school-age children in the 2007 Spring School census.  Clearly the majority of all movements occur within a 10km radius of the home.  Pane (b) shows the typical distances associated with primary and secondary schools, based on mean school-school distances.

We then consider an ODE model, based on POLYMOD, of a hospital catchment with a proportion of the population, F, in that catchment affected by an optimally timed single school closure of one week. The schematic representation of this model is shown in Pane (c) and its output in Pane (d).  We use the model to test how the reduction in epidemic peak scales with the size of school closures and assumptions about themovement and interaction of adults living in the school closure region. Throughout we assume that a fraction \begin{equation*}1-\varepsilon\end{equation*} of all adult interactions occur within the closure region, with the remainder occuring outside. Shown inblue is the simplest assumption that these external interactions are to outside the hospital catchment area and have no dynamical impact on the external population. The alternative assumption (shown in red) is that all of the interactions are within the hospital catchment area, and therefore have a dynamical impact on the total peak within that region. It is worth noting that a smaller fraction of school closures within the hospital catchment area necessiates a later closure to achieve the maximum impact on the total peak.

### Sensitivity to hospital catchments

We also consider the validity of assumptions made in our model about hospital catchments.  One assumption was to ignore the dynamical effects of heterogeneity in age profiles between hospital catchment areas (although we do consider the variability in adult numbers relative to capacity). This is shown to be reasonable by Pane (a) of Figure S3. A second assumption is that individuals move to their nearest hospital in the first instance. We can test the potential impact of alternative protocols by assuming that the movement of individuals minimises the quantity

\begin{equation*}\frac{\text{Distance}}{(\text{Total Beds})^\alpha}\end{equation*}

and then finding a value for α that minimises the number of adults over capacity in the absence of school closures, with results shown in Pane (b). Taking the optimal value of \begin{equation*}\alpha = 0.7\end{equation*} for the baseline of 100% of national capacity does not have a particularly dramatic effect even when school closures are included.  Panes (c) and (d)  of Figure S3 correspond to the default \begin{equation*}\alpha = 0\end{equation*} (as in the main text), while Panes (e) and (f) show how this changes when \begin{equation*}\alpha = 0.7\end{equation*}.

### A typical location

To demonstrate intuitively the complexities involved at a local scale, we show a typical area around a hospital in Figure S4. In this figure, the hospital location is shown as a grey marker, surrounded by the convex hull around its catchment. Movements from pupil home locations to school locations are shown as lines coloured by school; these are extremely dense within the (approximate) catchment region and extend a significant distanceoutside it, demonstrating the complex structure of interactions even at a local scale.

### Sources of data

Our work draws on a large number of data sources, which we detail below:

**Digital boundary data**

Source: 2001 Census, Output Area Boundaries.  Crown copyright 2003. Office for National Statistics, 2001 Census: Digitised Boundary Data (England and Wales) [computer file]. ESRC/JISC Census Programme, Census Geography Data Unit (UKBORDERS), EDINA (University of Edinburgh).  General Register Office for Scotland, 2001 Census: Digitised Boundary Data (Scotland) [computer file]. ESRC/JISC Census Programme, Census Geography Data Unit (UKBORDERS), EDINA (University of Edinburgh). Census output is Crown copyright and is reproduced with the permission of the Controller of HMSO and the Queen's Printer for Scotland.**Super Output Area boundaries** Source: Office for National Statistics, Super Output Area Boundaries. Crown copyright 2004. Crown copyright material is reproduced with the permission of the Controller of HMSO.**Super Output Area lookup tables** Source: Office for National Statistics, Neighbourhood Statistics DVD-ROM V1.3 (April 2005).**Super Output Area populations** Source: Office for National Statistics, Mid-2007 Population Estimates for Lower Layer Super Output Areas in England and Wales by Broad Age Group and Sex. Crown copyright 2008.**Hospital Capacity** Source: Department of Health form KH03a, Open and staffed adult critical care beds at 15 January 2009, by location and level of care, NHS Trusts in England. Published 27 February 2009.**Hospital Locations** Source: www.nhs.uk/servicedirectories/Pages/AcuteTrustListing.aspx **Geographical lookup** Provided by GeoConvert: geoconvert.mimas.ac.uk **School data** Source: Department for Children, Schools and Families, PLASC/Census 2006/2007 Spring data.**Interaction data** Source: 2001 Census: Special Workplace Statistics (England, Wales and Northern Ireland). Office for NationalStatistics, 2001 Census: Special Workplace Statistics (England, Wales and Northern Ireland) [computer file]. ESRC/JISC Census Programme, Centre for Interaction Data Estimation and Research (University of Leeds). Census output is Crown copyright and is reproduced with the permission of the Controller of HMSO and the Queen's Printer for Scotland.**H1N1 incidence** Source: Department of Health. Q-Surveillance(R) and National Pandemic Flu Service data.

### Supplementary Figures

**Figure:**
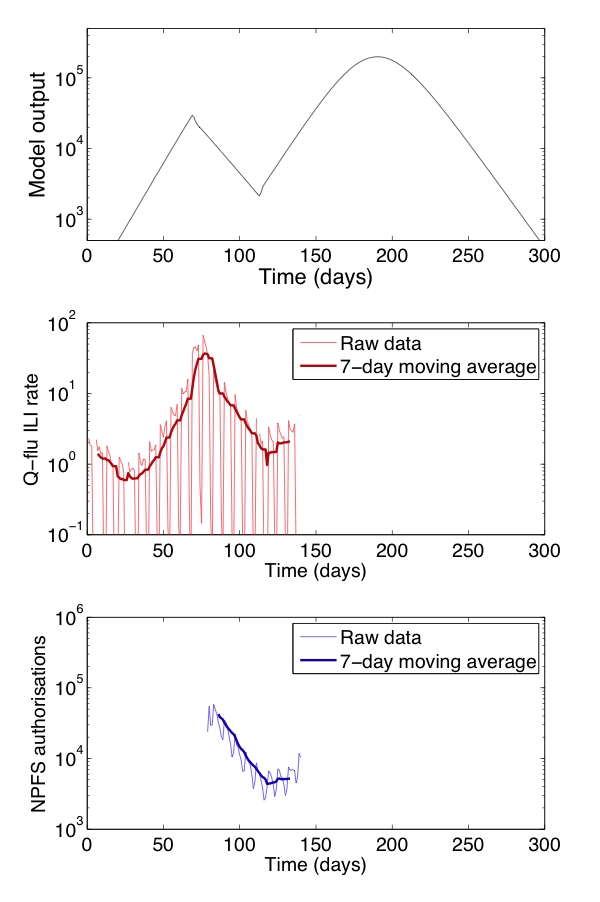
Figure S1: Results from the dynamical model used to consider school closure timings, compared to Q-flu ILI consultations and NPFS authorisations.

**Figure:**
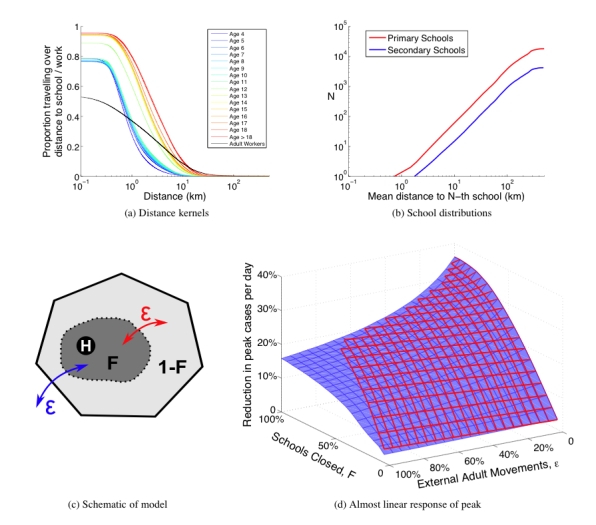
Figure S2: Geographical scale of (a) commuter movements, school movements, and (b) school locations.  (c) shows a schematic of the model used to test linearity of response to school closure - a hospital H has a catchment (light grey region) with a fraction F of that catchment subject to school closure. We then let a fraction \begin{equation*}\varepsilon\end{equation*} of adults interact with either the remainder of the hospital catchment (red) or the rest of England (blue).  (d) presents modelling results showing the approximate linearity of response of peak reduction with percentage of schools closed for within-catchment (red) and rest-of-England (blue) interactions across all spatial parameters.

**Figure:**
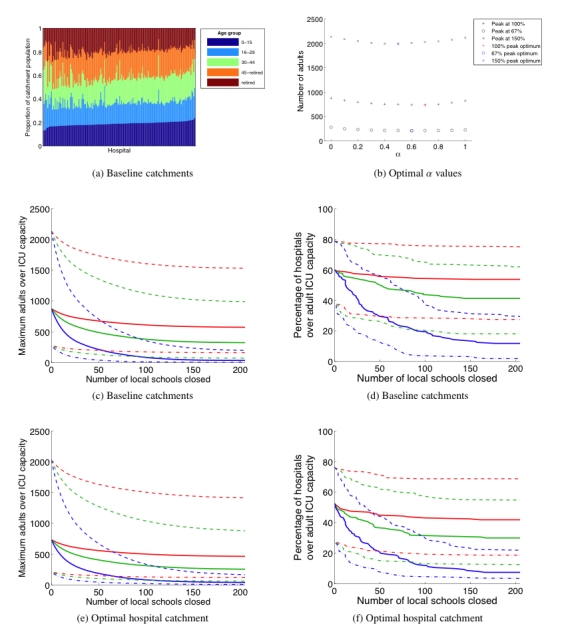
Figure S3: Features of and modification to hospital catchments.  (a) Baseline catchment area demographics, ordered by the proportion of children in the catchment area.  (b) Allocating LSOAs to hosptials on the basis of minimising \begin{equation*}\mathrm{distance} / \mathrm{beds}^{\alpha}\end{equation*} (baseline \begin{equation*}\alpha =0\end{equation*}) then optimising α to minimise the number of adults per adult ICU bed in the catchment. The baseline results (c) and (d) are modified to (e) and (f) at this optimum, which does not therefore represent a significant difference.

**Figure:**
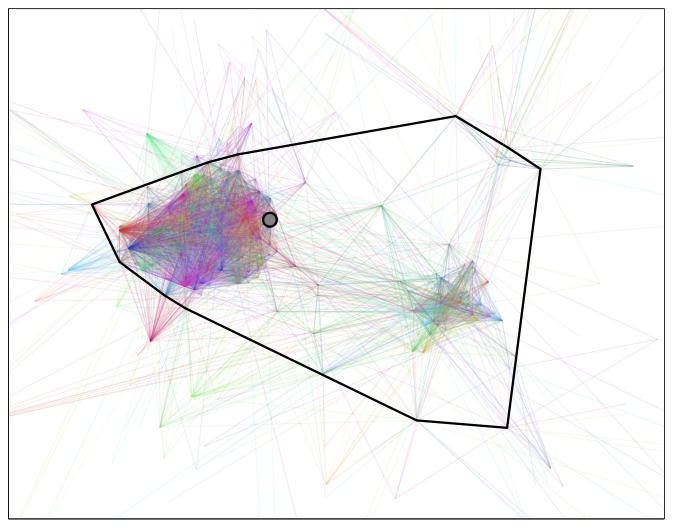
Figure S4: A typical hospital, shown in as a grey circle with a black border. The convex hull around the population-weighted centroids of LSOAs in the catchment area is shown as a black line, to avoid problems of identifiability with exact boundaries. Links between pupil home and school, granulated at the level of LSOA centroids, are shown as lines coloured by school.
